# Morphometric parameters of muscle and bone in critically ill patients: Post hoc analysis of the VITdAL-ICU trial

**DOI:** 10.1007/s00508-020-01736-4

**Published:** 2020-09-18

**Authors:** Oliver Malle, Dietmar Maurer, Doris Wagner, Christian Schnedl, Steven Amrein, Thomas Pieber, Astrid Fahrleitner-Pammer, Hans Peter Dimai, Karin Amrein

**Affiliations:** 1 Department of Internal Medicine, Division of Endocrinology and Diabetology, Medical University of Graz, Graz, Austria; 2 Department of Surgery, Medical University of Graz, Graz, Austria; 3 Department of Interventional and Diagnostic Radiology, General Hospital of Klagenfurt, Graz, Austria; 4 Department of Anesthesiology, Elisabethinen Hospital, Graz, Austria

**Keywords:** Sarcopenia, Intensive care, Mortality, Total psoas area, Skeletal muscle index

## Abstract

**Background:**

Sarcopenia, defined as loss of muscle mass, quality and function, is a part of the frailty syndrome. In critical illness, sarcopenia has rarely been evaluated regarding clinical outcomes. Therefore, we evaluated the association of sarcopenia with both hospital length of stay (HLOS) and 6‑month mortality in critically ill patients using abdominal computed tomography (CT) scans.

**Methods:**

In a post hoc analysis from the high dose vitamin D3 vs. placebo in adult vitamin D deficient patients (VITdAL-ICU) trial, we retrospectively reviewed all available abdominal CT scans (18 women, 19 men). We measured and calculated total psoas area (TPA), psoas muscle density (PMD), skeletal muscle index (SMI) and bone mineral density (BMD) and analyzed the relation of these endpoints with HLOS and mortality. Defining sarcopenia we used cut-off values for TPA as 642.1 mm^2^/m^2^ in women and 784 mm^2^/m^2^ in men and PMD as 31.1 Hounsfield units (HU) in women and 33.3 HU in men, both measured at the level of L3, as well as for SMI (38.5 cm^2^/m^2^ in women and 52.4 cm^2^/m^2^ in men). Likely osteoporosis was defined by L1 trabecular attenuation of ≤110 HU. Values for TPA, PMD and SMI could not be obtained in 11 patients and BMD in 1 patient.

**Results:**

Mean adjusted TPA was lower in women versus men (478 vs. 749 mm^2^/m^2^) as well as PMD (34.6 vs. 41.3 HU), SMI (62.36 vs. 76.81 cm^2^/m^2^) and BMD (141.1 vs. 157.2 HU). No significant influence on hospital length of stay and on 6‑month mortality was found, irrespective of the morphometric parameter used (TPA, PMD, SMI, BMD; *p* > 0.05). Survivors showed statistically nonsignificantly better values than nonsurvivors: TPA: 652 vs. 530 mm^2^/m^2^ (*p* = 0.27); PMD: 38.4 vs. 37.4 HU (*p* = 0.85); SMI: 70.32 vs. 69.54 cm^2^/m^2^ (*p* = 0.91); BMD: 156 vs. 145.8 HU (*p* = 0.81).

**Conclusion:**

Although the study is limited by the small sample size, our data do not support a strong predictive value for TPA/PMD/SMI or BMD for HLOS or mortality in critically ill patients with vitamin D deficiency.

## Introduction

Sarcopenia, characterized as reduction in skeletal muscle mass and strength, can compromise physical function and quality of life. In the literature there are heterogenous definitions of sarcopenia: however, a worldwide widely used definition was proposed in 2010 by the European Working Group on Sarcopenia in Older People (EWGSOP), which takes into account low muscle strength as a key characteristic [1]. Criteria used in other proposed definitions were low skeletal muscle mass either alone or with low muscular function assessed by grip strength and/or slow gait speed [2]. Depending on the criteria used for the definition, the reported prevalence of sarcopenia varies significantly. Based on dual-energy X‑ray absorptiometry (DXA), derived measurements adjusted to body height, the prevalence of sarcopenia was reported to be 53% in men and 43% in women at the age of >80 years [3]. Assessing sarcopenia with standard computed tomography (CT) scans is easy in theory. Millions of CT scans are performed yearly carrying potentially useful information about sarcopenia. Retrieval of data available on body CT examinations performed for any indication requires no additional cost, patient time, equipment or radiation exposure and can also be acquired retrospectively. In the literature, there is heterogeneity about the impact of sarcopenia on mortality. Some published data suggested an independent association of sarcopenia with increased mortality critical illness [4, 5]. Besides total psoas area (TPA), we evaluated qualitative parameters including psoas muscle density (PMD) and bone mineral density (BMD) as well as the skeletal muscle index (SMI).

The objective of the present analysis was to evaluate the ability of these morphometric parameters (TPA, PMD, BMD and SMI) in predicting 6‑month mortality and hospital length of stay (HLOS) in a cohort of mixed vitamin D deficient patients.

## Methods

This evaluation is a retrospective analysis based on the high dose vitamin D3 vs. placebo in adult vitamin D deficient patients (VITdAL-ICU) clinical trial, which originally included 480 critically ill patients with vitamin D deficiency (defined as a 25-hydroxyvitamin D < 20 ng/ml) [6]. A routine abdominal CT scan for any indication was available in 37 patients between 6 months before and 3 days after intensive care unit admission. A total of 11 CT examinations could not be included in some analyses due to missing scan levels of L3.

The scans were exported from the hospital radiology system as DICOM files. The OsiriX 8.0 software (Pixmeo SARL, Geneva, Switzerland) was used to analyze the scans including measuring and quantifying the morphometric parameters. Clinicopathologic data including age, sex, weight, height, body mass index (BMI), admission diagnosis, survival time and HLOS were available from the original data set. The body surface area was computed using the formula by Mosteller $\{\text{body surface} [\text{m}^2]= \text{height} [\text{cm}] \times (\text{weight} [\text{kg}]/3600)\}$ . In order to divide the patient cohort into a sarcopenia and non-sarcopenia group (similarly for BMD: likely and unlikely osteoporosis group) we used predefined cut-off values for morphometric parameters based on previous published data [7]. Furthermore, the study group was also separated into survivors (surviving longer than 6 months after ICU admission) and non-survivors (death within 6 months after ICU admission).

### Morphometric parameters

#### Measurement of TPA at the level of L3

**Fig. 1:**
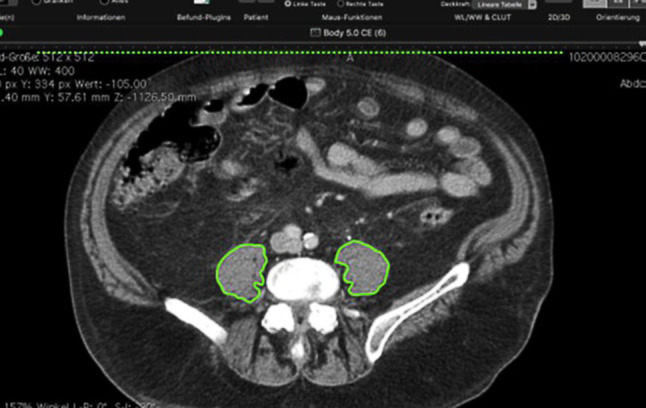
Cross-sectional X-ray image at the L3 level showing measurement of TPA in the psoas muscles (marked *green*)

The TPA was assessed by measuring the area of the right and left psoas muscles at the defined level of L3, where both iliac crests were clearly visible, by hand tracing the muscle borders manually and setting the density threshold between −29 Hounsfield units (HU) and 150 HU (Fig. 1). The measurements were normalized to the body surface. Assessing sarcopenia, the predefined TPA cut-off value was 642 mm^2^/m^2^ for women and 784 mm^2^/m^2^ for men [7].

#### Measurement of PMD at the level of L3

The skeletal muscle attenuation coefficient was used to assess the muscle quality, represented by PMD. With PMD, the lipid contained within skeletal muscle is quantified on the basis of attenuation characteristics, containing information about the tissue density and chemical composition [8]. Attenuation values are expressed in HU on the basis of a linear scale using water as the reference (0 HU). The cut-off values for defining sarcopenia in PMD were 31.1 HU in women and 33.3 HU in men [9].

#### Measurement of SMI at the level of L3

**Fig. 2:**
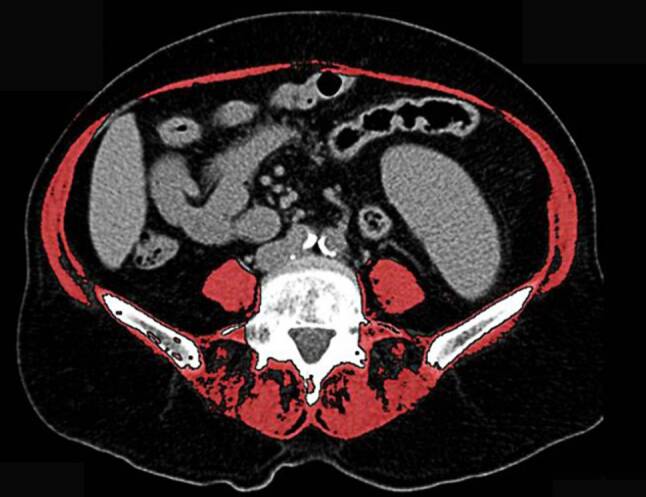
Cross-sectional X-ray image at the L3 level showing measurement of SMI (marked *red*)

Assessing sarcopenia with SMI, the cross-sectional area of all muscles at the L3 level was measured (Fig. 2). It is reported to be linearly related to whole body muscle mass [10]. It was then adjusted to body surface (= skeletal muscle index, cm^2^/m^2^). Sex-specific cut-off values more than two standard deviations below that of healthy adults were described elsewhere [11] and used in our analysis as well. These values are 38.5 cm^2^/m^2^ for women and 52.4 cm^2^/m^2^ for men.

#### Measurement of BMD at the level of L1

**Fig. 3:**
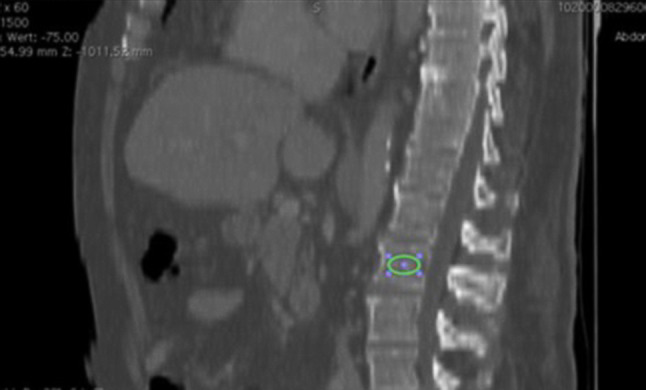
Sagittal cross-sectional X-ray image showing measurement of BMD at L1 (marked *green*)

**Fig. 4:**
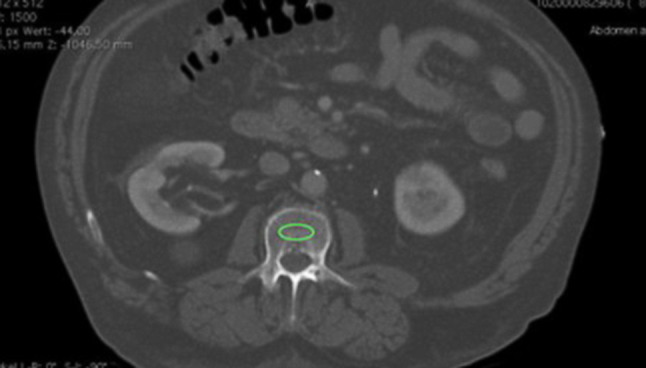
Cross-sectional X-ray image showing measurement of BMD at L1 (marked *green*)

Similar to PMD, BMD was assessed by measuring the CT attenuation at L1 by placing an oval region over an area of the anterior trabecular bone of the vertebral body. The measurements were performed in both the axial and the sagittal plane (Fig. 3 and 4). A lower attenuation expressed by lower HU represents less dense bone and indicates the probable presence of osteoporosis [12]. We chose level L1 due to the easy identification and presence in abdominal as well as thoracic CT examinations in routine practice. Areas such as the posterior venous plexus and conditions leading to distortion of BMD were avoided.

According to the abovementioned cut-off values, patients were divided into a sarcopenia and a no sarcopenia group for further analyses. Furthermore, the cohort was divided into survivors and non-survivors as well.

### Statistical analysis

Data are presented as mean and standard deviation (SD) for continuous variables. Independent sample t‑tests were used to compare the subgroups of the cohort (sarcopenia versus non-sarcopenia group, likely versus unlikely osteoporosis group, survivor versus non-survivor group) with normal distributions. For the assessment of mortality rates in the sarcopenia and non-sarcopenia group as well as in the likely and unlikely osteoporosis group we used the χ^2^-test and Yates’ correction due to the limited data to prevent overestimation of statistical significance. For determining the correlation between the morphometric parameter and patient age, Pearson correlation coefficient was used. A *p*-value of less than 0.05 was considered statistically significant. The statistical software package used was IBM® SPSS® Statistics Version 23 (IBM SPSS Statistics for Windows, Version 23.0, Released 2015, IBM Corp., Armonk, NY, USA).

## Results

The mean age was 58.8 ± 17.3 years, 18 (49%) were women and 19 (51%) were men. The mean BMI value was 26 kg/m^2^ and higher in women than in men. The analyzed population presented a broad variety of admission diagnoses, including neurologic (27%), gastrointestinal (18.9%) and others (Table 1), 57% of the patients were given artificial respiration, 40% were receiving vasopressor agents.

**Table 1:** Clinical characteristics, values of the morphometric parameters and percentage of patients with sarcopenia assessed with the respective parameters

	All patients	Men	Women	*p* value
*Number*	37	19 (51%)	18 (49%)	–
Age at ICU admission	58.8 ± 17.3	55.7 ± 18.6	62.1 ± 15.6	0.26
*Admission diagnosis*				
Neurological	10	4	6	–
Gastrointestinal	7	3	4	–
Cardiovascular	6	3	3	–
Traumatic	6	5	1	–
Respiratory	6	3	3	–
Infection (Sepsis, Abscess)	2	1	1	–
BMI (kg/m^2^)	26.0 ± 4.4	25.4 ± 4.0	26.6 ± 4.8	0.41
TPA (mm^2^/m^2^)	624 ± 233	749 ± 225	478 ± 145	0.002
PMD (HU)	38.2 ± 11.8	41.3 ± 14.1	34.6 ± 7.6	0.14
SMI (cm^2^/m^2^)	70.14 ± 14.79	76.81 ± 14.01	62.36 ± 11.92	0.01
BMD (HU)	149.2 ± 55.1	157.2 ± 51.9	141.1 ± 58.5	0.41
*Sarcopenia assessed*				
By TPA	*n* = 18 (69%)	*n* = 8 (57%)	*n* = 10 (83%)	
By PMD	*n* = 9 (35%)	*n* = 6 (43%)	*n* = 3 (25%)	
By SMI	*n* = 1 (4%)	*n* = 1 (14%)	*n* = 0 (0%)	
*Osteoporosis assessed*				
By BMD	*n* = 10 (28%)	*n* = 4 (22%)	*n* = 6 (33%)	

*ICU* intensive care unit, *TPA* total psoas area, *PMD* psoas muscle density, *SMI* skeletal muscle index, *BMD* bone mineral density

**Fig. 5:**
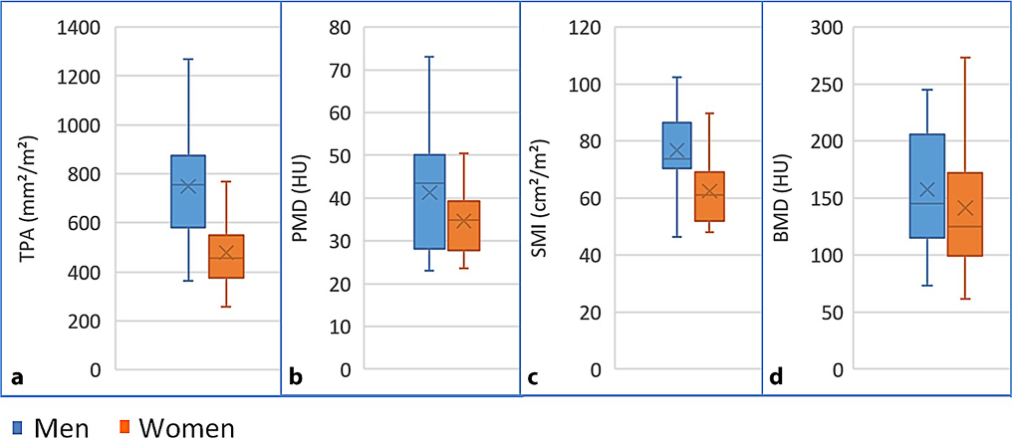
Distribution of **a** TPA, **b** PMD, **c** SMI and **d** BMD in men and women

After adjusting to body surface, the mean TPA was 624 mm^2^/m^2^. When stratified by gender, women showed significantly lower TPA (478 vs. 749 mm^2^/m^2^, *p* = 0.002) as well as significantly lower SMI (62.4 vs. 76.8 cm^2^/m^2^, *p* = 0.01) than men. The analysis of the other morphometric parameters showed numerically similar results but without statistical significance and are outlined in Table 1. The prevalence of sarcopenia significantly varied depending on the criteria used for definition. Based on TPA, sarcopenia was present in 69% of patients. Defined by PMD and SMI, the percentage of patients presenting with sarcopenia was lower (35% and 4%, respectively). The differences between women and men are summarized in Table 1 and Fig. 5. Using the predefined cut-off values for SMI, there is only one single patient classified as sarcopenic. This should be considered when interpreting the results relating to SMI.

**Fig. 6:**
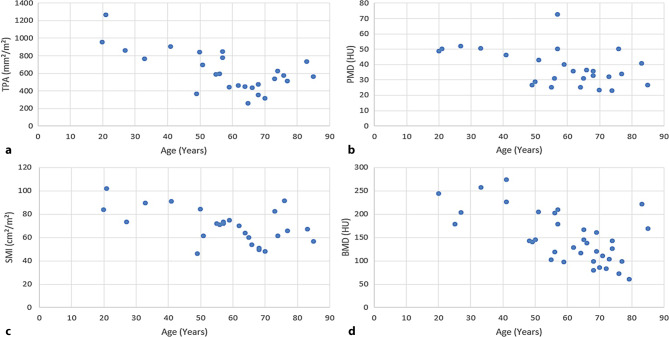
Distribution of **a** TPA, **b** PMD, **c** SMI and **d** BMD among age

As expected, all parameters showed a strong negative association with age (TPA: r = −0.67, *p* < 0.001; PMD: r = −0.46, *p* = 0.01; SMI: r = −0.53, *p* < 0.003; BMD: r = −0.62, *p* < 0.001) (Fig. 6).

The mean length of hospital stay was 13 days (interquartile range 7.5–24 days) and did not differ significantly among patients with or without sarcopenia defined by TPA (15.0 vs. 18.1 days; *p* = 0.49), PMD (13.4 vs. 17.3 days; *p* = 0.38) and BMD (14.0 vs. 18.6 days; *p* = 0.34) (Table 2). The 6‑month mortality was similar in patients with and without sarcopenia, irrespective of the morphometric parameter used (Table 3). As mentioned above, the results of SMI in sarcopenia and non-sarcopenia group (hospital length of stay: 31.0 vs. 15.36 days, *p* = 0.14; mortality rate: 100% vs. 20%, *p* = 0.52) were not meaningful, as there was just one patient classified as sarcopenic. Assessing TPA, PMD and SMI 11 patients were excluded due to missing scan levels at L3. Assessing BMD one patient was excluded due to vertebral fractures.

**Table 2:** Comparison of hospital length of stay between sarcopenia and non-sarcopenia patients

Hospital length of stay [days]	Sarcopenia	No sarcopenia	*p* value
Assessed by* TPA*	15.0 ± 10.9	18.1 ± 9.0	0.49
Assessed by* PMD*	13.4 ± 10.3	17.3 ± 10.3	0.38
Assessed by* SMI*	31.0 ± 0	15.36 ± 10.02	0.14
–	*Likely osteoporosis*	*No likely osteoporosis*	–
Assessed by* BMD*	14.0 ± 8.6	18.6 ± 13.7	0.34

*TPA* total psoas area, *PMD* psoas muscle density, *SMI* skeletal muscle index, *BMD* bone mineral density

**Table 3:** Comparison of 6‑month mortality between sarcopenia and non-sarcopenia patients

6‑month mortality	Sarcopenia	No sarcopenia	*P* value^a^
Assessed by* TPA*	28% (*n* = 5/18)	13% (*n* = 1/8)	0.73
Assessed by* PMD*	22% (*n* = 2/9)	24% (*n* = 4/17)	1.0
Assessed by* SMI*	100% (*n* = 1/1)	20% (*n* = 5/25)	0.52
–	*Likely osteoporosis*	*No likely osteoporosis*	–
Assessed by* BMD*	30% (*n* = 3/10)	31% (*n* = 8/26)	1.0

*TPA* total psoas area, *PMD* psoas muscle density, *SMI* skeletal muscle index, *BMD* bone mineral density

^a^With Yates’ correction

Morphometric parameters were not significantly better in survivors (*n* = 26) compared to non-survivors (*n* = 11) (TPA: 652 vs. 530 mm^2^/m^2^, *p* = 0.27; PMD: 38.4 vs. 37.4 HU, *p* = 0.85; SMI: 70.3 vs. 69.5 cm^2^/m^2^, *p* = 0.91; BMD 151 vs. 146 HU, *p* = 0.81) (Table 4).

**Table 4:** Comparison of the morphometric data in survivors and non-survivors

	Survivors	Non-survivors	*P* value
*TPA* (mm^2^/m^2^)	652 ± 239	530 ± 204	0.27
*PMD *(HU)	38.4 ± 12.8	37.4 ± 8.9	0.85
*SMI *(cm^2^/m^2^)	70.32 ± 13.03	69.54 ± 21.14	0.91
*BMD *(HU)	151 ± 54.4	145.8 ± 59.2	0.81

*TPA* total psoas area, *PMD* psoas muscle density, *SMI* skeletal muscle index, *BMD* bone mineral density

## Discussion

Frailty can be assessed in a number of ways but it appears attractive to use novel but simple analytical methods to assess sarcopenia and bone mineral density with routine CT images. While it was traditionally assessed by only one single cross-sectional measurement of the psoas muscle (TPA) [13], recently several other morphometric parameters (PMD, SMI, BMD) have increasingly been used. Obtaining a predictive marker for prognosis of critically ill patients is of interest as it may improve the risk stratification of critically ill patients.

In our small, retrospective analysis of the VITDAL-ICU trial, we aimed to assess morphometric parameters in all available CT scans and test if there was a strong predictive value of any of these on important clinical outcomes.

All morphometric parameters diminished with increasing age, with the strongest effect noted for TPA. As expected, men showed significantly higher values than women for TPA, PMD, SMI and BMD. These results confirmed that age and sex should be taken into account in the definition of sarcopenia. The morphometric parameters TPA, PMD, SMI and BMD were not significantly associated with both hospital length of stay and 6‑month mortality rate; however, all results were numerically better in survivors. Given that our sample size was very small, we cannot exclude a type 2 error in statistical analysis and that results of a bigger cohort may become significant. Currently, the results in the literature are conflicting [4, 14–16]. Notably, we did not evaluate the influence of volumetric parameters (i.e., total psoas volume), that have been shown to be associated with patient mortality and hospital length of stay [17]. Several limitations of the present analysis should be considered. Due to the low number of patients, results should be interpreted with caution. Also, CT examinations were not performed at a specific time point due to the retrospective nature of this study. For the same reason, it was not possible to measure other frailty parameters, for example grip strength. Because of the use of routine CT examinations, unenhanced and contrast-enhanced images were both analyzed. Thus, errors in measuring attenuation values could not be excluded. Furthermore, comorbidities such a malignant diseases or medication that may affect bone density were not assessed and may therefore distort results.

Defining sarcopenia obtained by morphometric parameters in CT scans is also unable to include muscle function. As there is no linear connection between muscle mass and muscle function [18], both should be included for the diagnosis of sarcopenia. As this is, however, impossible in ventilated or severely ill patients, the identification of a morphologic surrogate parameter could be of interest.

In conclusion, sarcopenia assessed with morphometric parameters obtained from CT scans was not an independent predictive factor of hospital length of stay and 6‑month mortality in critically ill, vitamin D deficient patients. The validity of these results is limited due to the relatively small patient cohort of 37.
